# Ethanol from Sorghum

**DOI:** 10.1186/1753-6561-8-S4-O21

**Published:** 2014-10-01

**Authors:** André Junqueira Luiz Franco

**Affiliations:** 1 CERES, CREA, São Paulo, São Paulo, 05026-001, Brazil

## 

Ceres Sementes do Brasil, Ltda is an affiliate of Ceres, Inc, founded in 1996 with headquarters in Thousand Oaks, California, USA, focused on development and marketing of non-food grasses for advanced biofuels and biopower with the aim of providing new opportunities for growers and a cleaner environment for us all. In 2009, Ceres Inc. founded its first Brazilian affiliate in São Paulo and initiated research in Brazil aiming to prepare the Brazilian market form commercialization of sweet sorghum and high biomass sorghum hybrids developed for production of biofuel and bioenergy. Ceres works directly with leading mills and suppliers of biofuel technologies to facilitate the introduction of sweet sorghum into existing operations and infrastructure and also carry out tests with leader institutions and innovative producers. Ceres current products are commercialized under the brand Blade^® ^and plans to introduce a number of high-yielding sorghum hybrids with high biomass and high sugar content to complement or expand feedstock supplies in sugarcane-growing regions. In addition to a conventional breeding program, Ceres is developing genetically modified seeds, improved for productivity and agronomic characteristics. We hold a wide collection of plant genes that we plan to introduce in our sorghum breeding program in Brazil.

The presentation will cover some considerations about the opportunities for sweet sorghum as a complementary alternative to sugarcane for production of ethanol, electric energy and, in the future, sugar production. It will also discuss some perspectives related to high biomass sorghum as a current alternative to complement biomass production before the sugarcane crop and potential markets in pellet production, heat/vapor and second generation ethanol. The challenges to the adoption of these alternative crops and to the development of new conventional as well as genetically modified hybrids will be addressed.

